# The impact of statin use on pneumonia risk and outcome: a combined population-based case-control and cohort study

**DOI:** 10.1186/cc11418

**Published:** 2012-07-12

**Authors:** Anders Gunnar Nielsen, Rikke Beck Nielsen, Anders Hammerich Riis, Søren Paaske Johnsen, Henrik Toft Sørensen, Reimar Wernich Thomsen

**Affiliations:** 1 Department of Clinical Epidemiology, Aarhus University Hospital, 8200 Aarhus N, Denmark

## Abstract

**Introduction:**

The impact of statin use on pneumonia risk and outcome remains unclear. We therefore examined this risk in a population-based case-control study and did a 5-year update of our previous 30-day mortality analyses.

**Methods:**

We identified 70,953 adults with a first-time hospitalization for pneumonia between 1997 and 2009 in Northern Denmark. Ten age- and sex-matched population controls were selected for each pneumonia patient. To control for potential confounders, we retrieved individual-level data on other medications, comorbidities, recent surgery, socioeconomic indicators, influenza vaccination, and other markers of frailty or health awareness from medical databases. We followed all pneumonia patients for 30 days after hospital admission.

**Results:**

A total of 7,223 pneumonia cases (10.2%) and 64 523 controls (9.1%) were statin users before admission, corresponding to an age- and sex-matched odds ratio (OR) of 1.17 (95% confidence interval [CI]: 1.14-1.21). After controlling for higher comorbidity and a wide range of other potential confounders, the adjusted OR for pneumonia associated with current statin use dropped to 0.80 (95% CI: 0.77-0.83). Previous statin use was not associated with decreased pneumonia risk (adjusted OR = 0.97, 95% CI: 0.91-1.02). Decreased risk remained significant after further adjustment for frailty and health awareness markers.

The prevalence of statin use among Danish pneumonia patients increased from 1% in 1997 to 24% in 2009. Thirty-day mortality following pneumonia hospitalization was 11.3% among statin users versus 15.1% among nonusers. This corresponded to a 27% reduced mortality rate (adjusted hazard ratio = 0.73, 95% CI: 0.67-0.79), corroborating our earlier findings.

**Conclusions:**

Current statin use was associated with both a decreased risk of hospitalization for pneumonia and lower 30-day mortality following pneumonia.

## Introduction

In addition to having a well-known cholesterol-lowering effect, statins regulate the immune and coagulation systems and prevent endothelial cell dysfunction [1-4]. These beneficial pleiotropic effects may explain why statin use has been associated with decreased risk and improved outcome of sepsis and other severe bacterial infections in a range of observational studies [5-9]. However, studies have reported conflicting results regarding the association between statin use and pneumonia risk. Although four studies reported a reduced risk [10-13], one US study showed an increased risk and suggested that a 'healthy-user effect' in statin users may have accounted for the earlier results [14]. Regarding pneumonia prognosis, we previously reported a 31% lower mortality among statin users following pneumonia hospitalization in a population-based study [15]. These findings are in line with several other studies, including large population-based cohorts [16-22]. However, one Canadian study showed no reduction in mortality among statin users hospitalized for pneumonia [23], and a recent US study found that statin use did not protect against severe sepsis or mortality in hospitalized patients with pneumonia [24]. Evidence thus remains conflicting, leaving open the possibility of uncontrolled confounding by healthy-user effects in statin users [25]. Importantly, the prevalence of statin use in Denmark and elsewhere has increased dramatically in recent years, resulting in less selection of patients receiving statins.

Since large clinical trials that address this issue are still lacking [26], we conducted the hitherto largest population-based case-control study on statin use and risk of pneumonia, taking advantage of new high-quality Danish data to better control for confounders. We also updated previous 30-day pneumonia mortality analyses [15].

## Materials and methods

### Setting

We conducted this study in Northern Denmark, which has 1.8 million inhabitants (30% of Denmark's population). All citizens in Denmark are provided with universal tax-supported health care, which guarantees unfettered access to primary and secondary care that is free at the point of delivery and provides partial reimbursement for most prescribed medications, including statins. The unique Danish civil registration number permits cross-linking of individual-level data from different medical databases.

### Patients with pneumonia

We identified patients with pneumonia from the Danish National Registry of Patients (DNRP), which contains data on inpatient discharges from all non-psychiatric hospitals in Denmark since 1977 and on emergency department and hospital outpatient clinic visits since 1995. Each contact is associated with one primary diagnosis and one or more secondary diagnoses classified according to the eighth revision of the *International Classification of Diseases *(ICD-8) until the end of 1993 and 10th revision (ICD-10) thereafter [27]. We included a total of 70,953 patients who were at least 15 years old with a first-time hospitalization for pneumonia during the study period (1 January 1997 to 12 December 2009). We excluded patients who had lived in the study region for less than 12 months.

### Population controls

We used the Danish Civil Registration System (CRS) to select 10 population controls for each case; the controls were individually matched on age (birth year), gender, index date, and place of residence. The CRS has maintained vital statistics data, including date of birth, change of address, date of emigration, and exact date of death for the Danish population since 1968. We selected controls by using risk set sampling: each control had to be alive and at risk for a first pneumonia hospitalization on the day of admission (the index date) of the case to whom he or she was matched. In total, it was possible to match 70,914 pneumonia patients with 709,140 population controls for the risk analysis. In the updated cohort outcome analyses, all 70,954 patients with pneumonia were followed up for 30 days after hospital admission, with all-cause mortality as the outcome.

### Statin use

Statins are available only by prescription in Denmark. As in our previous cohort study, current statin use was defined as at least one filled prescription within 125 days of the pneumonia hospitalization/index date [15]. Persons who filled a prescription more than 125 days beforehand were classified as former statin users. The 125-day period was chosen to capture most current users; if an 80% to 100% compliance rate is assumed, few statin prescriptions are expected to last beyond 125 days [28]. The prescription database contains information - including civil registration numbers, type and amount of drugs dispensed, and prescription dates - on reimbursable drugs dispensed at all pharmacies in Northern Denmark. In-hospital drug data were not available to allow an evaluation of the effectiveness of inpatient statin treatment in the cohort study.

### Covariates

To control for comorbidities that may be associated with pneumonia, we obtained data from the DNRP on all previous hospital diagnoses for each individual since 1977. We obtained data on the 19 disease categories that comprise the Charlson comorbidity index [29-31]. The DNRP also provided information on hospital-diagnosed obesity, alcoholism-related disorders within 1 year of the index date, and surgical procedures performed within 3 months of the index date. In addition, we obtained data on the use of the following preadmission medications, which may reflect important underlying comorbidities and/or affect either risk or prognosis of pneumonia: immunosuppressive drugs and oral steroids within 1 year of admission, systemic antibiotic therapy within 3 months of admission (for outcome analyses only), concurrent use (within 125 days of the index date) of inhaled corticosteroid therapy, inhaled beta2 agonists, combination of inhaled steroids and beta2 agonists, beta blockers, low-dose aspirin, loop diuretics, thiazides, angiotensin-converting enzyme inhibitors, angiotensin receptor antagonists, digoxin, vitamin K antagonists, calcium channel blockers, nitrates, antipsychotics, non-steroidal anti-inflammatory drugs, and proton-pump inhibitors. For ICD and Anatomical Therapeutic Chemical prescription codes, see Additional files 1 and 2.

To control for socioeconomic confounding, we obtained data from the CRS on marital status (married, divorced or widowed, never married, or unknown) and urbanization (city with more than 100,000 inhabitants, provincial town with 10,000 to 99,999 inhabitants, or rural area with fewer than 10,000 inhabitants).

To further control for a possible healthy-user effect in statin users, we obtained information from the National Health Insurance Service Registry for the period of 1 January 2001 to 12 December 2009 (for details, see Additional file 3). This registry contains detailed information on all patient contacts with general practitioners (GPs) and specialist practitioners in the primary-care sector. We retrieved data on receipt of the current season's reimbursed influenza vaccine prior to the pneumonia index date and on the following markers of health awareness or frailty: GP preventive consultations and services, social medicine-related GP consultations, GP conversational therapy, application for reimbursement for chronic and terminal illness, and GP diagnosis and treatment of dementia.

### Statistical analysis

In our setting with a relatively low proportion of treated subjects and outcome events and many time-varying covariates, we chose an incidence case-control study design with risk set sampling instead of a cohort design to study pneumonia risk [32,33]. In the case-control study, we used conditional logistic regression to estimate the odds ratio (OR) for hospitalization with pneumonia among current and former users of statins in comparison with never users. The controls were matched on age, sex, place of residence, and index date. We adjusted for the following covariates: individual comorbidities, preadmission drugs (except for systemic antibiotic therapy), socioeconomic indicators, living with small children, and (for the subcohort of cases and controls for 2001-2009) influenza vaccination and other markers of health awareness or frailty. Because we used risk set sampling of controls, the ORs provided unbiased estimates of incidence rate ratios.

In the cohort study, we used Cox regression analysis to compute 30-day mortality rate ratios (MRRs), controlling for sex, age, place of residence as well as the same covariates used in the case-control analysis, except for exclusion of living with small children and inclusion of preadmission antibiotic therapy in the prognosis analysis model.

We repeated both risk and outcome analyses while stratifying on calendar time periods, comorbidity levels, age groups, and medical indication for statin use in the main model (1997-2009). The risk estimates associated with simvastatin, atorvastatin, pravastatin, and other statins were assessed separately. In the subcohort analysis for 2001-2009, we further restricted the analyses to patients who received the current year's influenza vaccine and had different markers of frailty and health awareness. In the stratified analyses, the matching was retained.

Finally, we examined a potential dose-response association by calculating the number of defined daily doses (DDDs) of statin received within 125 days of the index date for each patient. The adjusted ORs and MRRs were plotted against statin DDD percentiles. Data were obtained from Danish health-care registries, which are generally available to researchers; according to Danish law, use of these registries does not require informed consent. The study was approved by the Danish Data Protection Agency (record number 2009-41-3866).

## Results

### Case-control study of pneumonia risk

We identified 70,914 cases of patients hospitalized for pneumonia and 709,140 population controls. Current statin users comprised 7,223 (10.2%) of the cases and 64,523 (9.1%) of the controls. Among current statin users, 78% received simvastatin, 12% received atorvastatin, 5.5% received pravastatin, and 4.5% received other statins. Slightly more than half of the cases were male; their median age was 73 years, and two thirds were older than 65 years (Table 1, case-control study). Compared with controls, pneumonia cases were more likely to have comorbidities, particularly cardiovascular disease, chronic pulmonary disease, cancer, or recent surgery. Medication use was also much more common among cases than controls, particularly diuretics and other cardiovascular therapies and corticosteroids. However, influenza vaccination was only slightly more common among cases.

**Table 1:** Case-control study: characteristics of 70,914 hospitalized patients with pneumonia and 709,140 population controls in Denmark (1997-2009)

Characteristics at time of hospital admission	Pneumonia cases (n = 70,914)	Population controls (n = 709,140)	Total (n = 780,045)
Age, number (percentage)			
15-39 years	5,140 (7.3)	51,601 (7.3)	56,741 (7.3)
40-64 years	17,803 (25.1)	178,095 (25.1)	195,898 (25.1)
65-79 years	26,122 (36.8)	261,046 (36.8)	287,168 (36.8)
≥80 years	21,849 (30.8)	218,398 (30.8)	240,247 (30.8)
Sex, number (percentage)			
Female	33,278 (46.9)	332,780 (46.9)	366,058 (46.9)
Male	37,636 (53.1)	376,360 (53.1)	413,996 (53.1)
Calendar period, number (percentage)			
1997-2000	17,754 (25.0)	177,540 (25.0)	195,294 (25.0)
2001-2004	23,786 (33.5)	237,860 (33.5)	261,646 (33.5)
2005-2009	29,374 (41.4)	293,740 (41.4)	323,144 (41.4)
Preadmission hospital-diagnosed comorbidities, number (percentage)			
Conditions included in the Charlson index			
Previous myocardial infarction	6,704 (9.5)	39,573 (5.6)	46,277 (5.9)
Congestive heart failure	7,457 (10.5)	29,791 (4.2)	37,248 (4.8)
Peripheral vascular disease	6,100 (8.6)	28,615 (4.0)	34,715 (4.5)
Cerebrovascular disease	10,672 (15.0)	62,515 (8.8)	73,187 (9.4)
Dementia	1,581 (2.2)	9,343 (1.3)	10,924 (1.4)
Hemiplegia	472 (0.7)	1,133 (0.2)	1,605 (0.2)
Chronic pulmonary disease	14,457 (20.4)	44,225 (6.2)	58,682 (7.5)
Connective tissue disease	3,759 (5.3)	19,158 (2.7)	22,917 (2.9)
Peptic ulcer disease	6,198 (8.7)	34,861 (4.9)	41,059 (5.3)
Mild liver disease	1,299 (1.8)	3,671 (0.5)	4,970 (0.6)
Moderate to severe liver disease	386 (0.54)	861 (0.12)	1,247 (0.2)
Diabetes without end-stage organ damage	5,662 (8.0)	28,185 (4.0)	33,847 (4.3)
Diabetes with end-stage organ damage	2,978 (4.2)	11,478 (1.6)	14,456 (1.9)
Moderate to severe renal disease	2,714 (3.8)	8,523 (1.2)	11,237 (1.4)
Solid cancer	10,737 (15.1)	62,092 (8.8)	72,829 (9.3)
Metastatic solid cancer	2,043 (2.9)	4,174 (0.6)	6,217 (0.8)
Leukemia	744 (1.0)	1,267 (0.2)	2,011 (0.3)
Lymphoma	1,307 (1.8)	2,490 (0.4)	3,797 (0.5)
AIDS	88 (0.1)	87 (0.0)	175 (0.0)
Charlson comorbidity index score			
Low, 0	26,285 (37.1)	457,185 (64.5)	483,470 (62.0)
Medium, 1-2	28,627 (40.4)	197,723 (27.9)	226,350 (29.0)
High, ≥3	16,002 (22.6)	54,232 (7.7)	70,234 (9.0)
Additional comorbidity			
Alcoholism-related disorders	4,303 (6.1)	13,871 (2.0)	18,174 (2.3)
Obesity	2,869 (4.1)	12,515 (1.8)	15,384 (2.0)
Any surgical procedure within 90 days of index date	14,782 (20.8)	40,385 (5.7)	55,167 (7.1)
Preadmission medications within 125 days of index date, unless otherwise indicated			
Statins, current use	7,223 (10.2)	64,523 (9.1)	71,746 (9.2)
Statins, former use (>125 days)	1,908 (2.7)	14,102 (2.0)	16,010 (2.1)
Oral steroids (within 1 year of index date)	13,124 (18.5)	45,763 (6.5)	58,887 (7.6)
Other immunosuppressive drugs (within 1 year of index date)	1,347 (1.9)	4,789 (0.7)	6,136 (0.8)
Users of inhalation medications			
Beta2 agonists only	5,514 (7.8)	15,110 (2.1)	20,624 (2.6)
Corticosteroid therapy only	823 (1.2)	5,463 (0.8)	6,286 (0.8)
Corticosteroid therapy and beta2 agonists	9,119 (12.9)	25,835 (3.6)	34,954 (4.5)
Beta blockers	11,988 (16.9)	93,125 (13.1)	105,113 (13.5)
Low-dose aspirin	12,731 (18.0)	95,825 (13.5)	108,556 (13.9)
Loop diuretics	17,804 (25.1)	76,134 (10.7)	93,938 (12.0)
Thiazides	9,432 (13.3)	85,475 (12.0)	94,907 (12.2)
Angiotensin-converting enzyme inhibitors	9,309 (13.1)	73,417 (10.4)	82,726 (10.6)
Angiotensin II antagonists	4,598 (6.5)	46,242 (6.5)	50,840 (6.5)
Digoxin	6,071 (8.6)	32,810 (4.6)	38,881 (5.0)
Vitamin K antagonist	4,024 (5.7)	22,590 (3.2)	26,614 (3.4)
Calcium antagonists	10,466 (14.8)	84,077 (11.9)	94,543 (12.1)
Nitrates	6,384 (9.0)	34,384 (4.9)	40,768 (5.2)
Antipsychotics	4,902 (6.9)	21,013 (3.0)	25,915 (3.3)
Non-steroidal anti-inflammatory drugs	12,641 (17.8)	87,279 (12.3)	99,920 (12.8)
Proton-pump inhibitors	11,469 (16.2)	50,146 (7.1)	61,615 (7.9)
Markers of socioeconomic status, number (percentage)			
Marital status, number (percentage)			
Married	33,423 (47.1)	376,521 (53.1)	409,944 (52.6)
Never married	8,957 (12.6)	80,230 (11.3)	89,187 (11.4)
Divorced or widowed	28,348 (40.0)	250,837 (35.4)	279,185 (35.8)
Unknown	186 (0.3)	1,552 (0.2)	1,738 (0.2)
Living with small children	940 (1.3)	7,981 (1.1)	8,921 (1.1)
Urbanization of place of residence, number (percentage)			
Rural	14,138 (20.0)	143,517 (20.2)	157,655 (20.2)
Provincial town	37,909 (53.5)	384,034 (54.2)	421,943 (54.1)
City	18,867 (26.6)	181,589 (25.6)	200,456 (25.7)
Markers of frailty and health awareness, number (percentage)^a^			
Receipt of current year's influenza vaccine before index date	9,538 (17.9)	74,873 (14.1)	84,411 (14.4)
Preventive GP consultations and services	5,863 (11.0)	37,349 (7.0)	43,212 (7.0)
Social medicine-related GP consultations	15,118 (28.4)	59,686 (11.2)	74,804 (12.8)
GP conversational therapy	5,124 (9.6)	18,738 (3,5)	23,862 (4.1)
Application for reimbursement for chronic illness	2,703 (5.1)	5,958 (1.1)	8,661 (1.5)
Application for reimbursement for terminal illness	274 (0.5)	229 (0.04)	503 (0.1)
GP diagnosis and treatment of dementia	560 (1.1)	4,046 (0.8)	4,606 (0.8)

^a^Ascertained among 53,160 pneumonia cases who could be matched with 531,600 controls for 2001-2009. GP, general practitioner.

The unadjusted age- and sex-matched OR associating current statin use with pneumonia occurrence was 1.17 (95% confidence interval (CI) 1.14 to 1.21) (Table 2, case-control study). Adjustment for potential confounders reduced the OR to 0.80 (95% CI 0.77 to 0.83). No association was found between former statin use and the risk of pneumonia (adjusted OR = 0.97, 95% CI 0.91 to 1.02). The adjusted ORs associated with use of simvastatin, atorvastatin, and pravastatin were 0.80 (95% CI 0.77 to 0.83), 0.83 (95% CI 0.76 to 0.91), and 0.86 (95% CI 0.76 to 0.98), respectively. For the period of 2001-2009, we further adjusted for receipt of influenza vaccine and markers of frailty and health awareness. This step left the OR of pneumonia associated with current statin use virtually unchanged (adjusted OR = 0.77, 95% CI 0.74 to 0.80) (Table 2, case-control study), whereas the adjusted OR for former statin use was 0.93 (95% CI 0.87 to 0.98).

**Table 2:** Case-control study: preadmission statin use and risk of hospitalization for pneumonia in Denmark (1997-2009)

Statin use	Pneumonia cases (n = 70,914)	Population controls (n = 709,140)	**Crude**^a ^**OR** (95% CI)	**Adjusted**^b ^**OR** (95% CI)
Never	61,783 (87.1%)	630,515 (88.9%)	1.00 (reference)	1.00 (reference)
Former (>125 days before admission)	1,908 (2.7%)	14,102 (2.0%)	1.42 (1.35-1.50)	0.97 (0.91-1.02)
Current (≤125 days before admission)	7,223 (10.2%)	64,523 (9.1%)	1.17 (1.14-1.21)	0.80 (0.77-0.83)
Current (≤125 days before admission), supplementary adjusted model for 2001-2009	6,883 (13.0%)	61,450 (11.6%)	1.18 (1.15-1.21)	0.77 (0.74-0.80)

^a^Matched for age, sex, urbanization of place of residence, and hospitalization/index date. ^b^Adjusted for marital status, calendar period, living with small children, any recent surgical procedure, 20 different comorbidities, alcoholism-related conditions, and 18 different preadmission medications (Table 1). The supplementary model for 2001-2009 includes 53,160 pneumonia cases and 531,600 controls and is also adjusted for receipt of current year's influenza vaccine, dementia, social medicine-related consultations, application for reimbursement for chronic or terminal illness, conversational therapy, and general practitioner preventive consultations and services. CI, confidence interval; OR, odds ratio.

We adjusted for important confounders separately to evaluate their importance in current statin users (data not shown). Comorbidity (individual adjustment for the 19 disease categories in the Charlson index) appeared to be the most important confounder (comorbidity-only adjusted OR = 0.86, 95% CI 0.84 to 0.89). Adjusting only for the use of 18 different medications yielded an OR of 0.90 (95% CI 0.87 to 0.93). Adjusting only for markers of frailty and health awareness yielded an OR of 1.03 (95% CI 1.00 to 1.06).

### Cohort study of 30-day mortality after hospitalization for pneumonia

The prevalence of current statin use among patients with pneumonia increased dramatically from 1997 (1%) to 2009 (24%). Thus, 74% of all statin users in our 1997-2009 cohort were hospitalized after 2004, which was the last year of study in our previous mortality analysis [15].

Characteristics of statin users and non-users with pneumonia in the cohort study are presented in Table 3. Statin users were less likely to be at the extremes of age but had much more previous cardiovascular disease and diabetes. Of importance, they also had higher rates of chronic pulmonary disease and corticosteroid use, a similar prevalence rate of cancer, but less liver disease and alcoholism in comparison with non-users. Statin users were more likely to be married, receive influenza vaccinations, and have preventive consultations with GPs. Reimbursement rates for chronic or terminal illness or dementia were similar in statin users and non-users (Table 3, cohort study).

**Table 3:** Cohort study: characteristics of 70,953 patients hospitalized for pneumonia for the first time in Denmark according to statin use (1997**-**2009)

Characteristics at time of pneumonia admission	Current statin use (≤125 days) n = 7,223 (10.2%)	Former statin use (>125 days) n = 1,903 (2.7%)	Never statin use n = 61,827 (87.1%)	Total n = 70,953 (100%)
Age, number (percentage)				
15-39 years	43 (0.6)	19 (1.0)	5078 (8.2)	5,140 (7.2)
40-64 years	1,756 (24.3)	518 (27.2)	15,529 (25.1)	17,803 (25.1)
65-79 years	3,819 (52.9)	929 (48.8)	21,374 (34.6)	26,122 (36.8)
≥80 years	1,605 (22.2)	437 (23.0)	19,846 (32.1)	21,888 (30.9)
Sex, number (percentage)				
Female	3,047 (42.2)	855 (44.9)	29,389 (47.5)	33,291 (46.9)
Male	4,176 (57.8)	1,048 (55.1)	32,438 (52.5)	37,662 (53.1)
Calendar period, number (percentage)				
1997-2000	340 (4.7)	62 (3.3)	17,359 (28.0)	17,761 (25.0)
2001-2004	1,520 (21.0)	318 (16.7)	21,957 (36.0)	23,795 (33.5)
2005-2009	5,363 (74.3)	1,523 (80.0)	22,511 (36.4)	29,397 (41.4)
Preadmission hospital-diagnosed comorbidities, number (percentage)				
Conditions included in Charlson index				
Previous myocardial infarction	2,346 (32.5)	482 (25.3)	3,877 (6.2)	6,705 (9.5)
Congestive heart failure	1,443 (20.0)	358 (18.8)	5,661 (9.2)	7,462 (10.5)
Peripheral vascular disease	1,506 (20.9)	389 (20.4)	4,206 (6.8)	6,101 (8.6)
Cerebrovascular disease	2,030 (28.1)	495 (26.0)	8,151 (13.2)	10,676 (15)
Dementia	134 (1.9)	36 (1.9)	1,412 (2.3)	1,582 (2.23)
Hemiplegia	36 (0.5)	6 (0.3)	430 (0.7)	472 (0.7)
Chronic pulmonary disease	1,728 (23.9)	431 (22.7)	12,299 (19.9)	14,458 (20.4)
Connective tissue disease	441 (6.1)	134 (7.0)	3,185 (5.2)	3,760 (5.3)
Peptic ulcer disease	735 (10.2)	215 (11.3)	5,253 (8.5)	6,203 (8.4)
Mild liver disease	82 (1.1)	52 (2.7)	1,165 (1.9)	1,299 (1.8)
Moderate to severe liver disease	17 (0.2)	22 (1.2)	347 (0.6)	386 (0.5)
Diabetes without end-stage organ damage	1,505 (20.8)	392 (20.6)	3,766 (6.0)	5,663 (8.0)
Diabetes with end-stage organ damage	942 (13.0)	242 (12.7)	1,794 (2.9)	2,978 (4.2)
Moderate to severe renal disease	532 (7.4)	189 (9.9)	1,993 (3.2)	2,714 (3.8)
Solid cancer	1,114 (15.4)	384 (20.2)	9,242 (15)	10,740 (15.1)
Metastatic solid cancer	195 (2.7)	87 (4.6)	1,761 (2.9)	2,043 (2.9)
Leukemia	53 (0.7)	19 (1.0)	672 (1.1)	744 (1.1)
Lymphoma	98 (1.4)	53 (2.8)	1,157 (1.9)	1,308 (1.8)
AIDS	3 (0.0)	0 (0.0)	85 (0.1)	88 (0.1)
Charlson comorbidity index score				
Low, 0	1,052 (14.6)	290 (15.2)	24,966 (40.4)	26,308 (37.0)
Medium, 1-2	3,259 (45.0)	772 (40.6)	24,609 (39.8)	28,640 (40.4)
High, ≥3	2,912 (40.3)	841 (44.2)	12,252 (19.8)	16,005 (22.6)
Additional comorbidity				
Alcoholism-related disorders	352 (4.9)	135 (7.1)	3,813 (6.2)	4,300 (6.1)
Obesity	588 (8.1)	137 (7.2)	1,906 (3.1)	2,631 (3.7)
Any surgical procedure within 90 days of index date	1,970 (27.3)	518 (27.2)	12,298 (19.9)	14,786 (20.8)
Preadmission medications within 125 days of index date unless otherwise indicated, number (percentage)				
Immunosuppressive drugs (within 1 year of index date)	157 (2.2)	49 (2.6)	1,141 (1.9)	1,347 (1.9)
Oral steroids (within 1 year of index date)	1,459 (20.2)	436 (22.9)	11,230 (18.2)	13,125 (18.5)
Systemic antibiotic therapy (within 90 days of index date)	2,995 (41.5)	790 (41.5)	24,978 (40.4)	28,763 (40.5)
Users of inhalation medications				
Beta2 agonists only	509 (7.0)	153 (8.0)	4,854 (7.9)	5,516 (7.8)
Corticosteroid therapy only	86 (1.2)	18 (1.0)	719 (1.2)	823 (1.2)
Corticosteroids and beta2 agonists	1,105 (15.3)	257 (13.5)	7,757 (12.6)	9,119 (12.9)
Beta blockers	3,290 (45.6)	579 (30.4)	8,124 (13.1)	11,993 (16.9)
Low-dose aspirin	3,473 (48.1)	665 (35.0)	8,605 (13.9)	12,743 (18.0)
Loop diuretics	2,672 (37.0)	589 (30.1)	14,558 (23.6)	17,819 (25.1)
Thiazides	1,347 (18.7)	309 (16.2)	7,782 (12.6)	9,438 (13.3)
Angiotensin-converting enzyme inhibitor	2,512 (34.8)	462 (24.3)	6,339 (10.3)	9,313 (13.1)
Angiotensin II antagonists	1,273 (17.6)	289 (15.2)	3,037 (4.9)	4,599 (6.5)
Digoxin	671 (9.3)	151 (7.9)	5,252 (8.5)	6,074 (8.6)
Vitamin K antagonist	927 (12.8)	179 (9.4)	2,918 (4.7)	4,024 (5.7)
Calcium antagonists	2,261 (31.3)	467 (24.5)	7,742 (12.5)	10,470 (14.8)
Nitrates	1,658 (23.0)	293 (15.4)	4,436 (7.2)	6,387 (9.0)
Antipsychotics	397 (5.5)	115 (6.0)	4,392 (7.1)	4,904 (6.9)
Non-steroidal anti-inflammatory drugs	1,341 (18.6)	349 (18.3)	10,953 (17.7)	12,643 (17.8)
Proton-pump inhibitors	1,860 (25.8)	542 (28.5)	9,071 (14.7)	11,473 (16.2)
Markers of socioeconomic status, number (percentage)				
Marital status, number (percentage)				
Married	4,131 (57.2)	1,034 (54.3)	28,261 (45.7)	33,426 (47.1)
Never married	463 (6.4)	102 (5.4)	8,393 (13.6)	8,958 (12.6)
Divorced or widowed	2,614 (36.2)	764 (40.2)	25,005 (40.4)	28,383 (40.0)
Unknown	15 (0.2)	3 (0.2)	168 (0.3)	186 (0.3)
Urbanization of place of residence, number (percentage)				
Rural	1,235 (17.1)	283 (14.9)	11,921 (18.3)	12,809 (18.0)
Provincial town	4,054 (56.1)	1,055 (55.4)	34,083 (55.1)	39,192 (55.2)
City	1,934 (26.8)	565 (29.7)	16,453 (26.6)	18,952 (26.7)
Markers of frailty or health awareness, number (percentage)^a^				
Receipt of current year's reimbursed influenza vaccine before index date	2,267 (32.9)	519 (28.2)	6,760 (15.2)	9,546 (18.0)
Preventive GP consultations and services	1,867 (27.1)	528 (28.7)	3,472 (7.8)	5,867 (11.0)
Social medicine-related GP consultations	1,967 (28.6)	578 (31.4)	12,589 (28.3)	15,134 (28.5)
GP conversational therapy	780 (11.3)	237 (12.9)	4,108 (9.2)	5,125 (9.6)
Application for reimbursement for chronic illness	670 (9.7)	145 (7.9)	1,888 (4.3)	2,703 (5.1)
Application for reimbursement for terminal illness	28 (0.4)	21 (1.1)	225 (0.5)	274 (0.5)
GP diagnosis and treatment of dementia	85 (1.2)	29 (1.6)	446 (1.0)	560 (1.1)

^a^Ascertained among 53,192 pneumonia patients hospitalized in 2001-2009. GP, general practitioner.

Our updated outcome analyses showed that cumulative 30-day mortality rates following pneumonia hospitalization were 11.3% among statin users and 15.1% among non-users (Table 4, cohort study). Results of Cox regression analysis indicated a 27% decrease in pneumonia mortality associated with current statin use (adjusted hazard ratio (HR) = 0.73, 95% CI 0.67 to 0.79); this finding is similar to our earlier findings [15]. Supplementary analyses that included influenza vaccination and other markers of frailty and health awareness for the 2001-2009 period did not materially alter this estimate (adjusted HR = 0.76, 95% CI 0.70 to 0.83). In neither of these analyses was former statin use associated with mortality.

**Table 4:** Cohort study: preadmission statin use and prognosis within 30 days following hospitalization for pneumonia in Denmark (1997-2009)

Statin use	Pneumonia patients (n = 70,953)	Number of deaths	30-day mortality, percentage	Crude HR (95% CI)	**Adjusted**^a ^**HR** (95% CI)
Never	61,827	9,282	15.1	1.00 (reference)	1.00 (reference)
Former (>125 days)	1,903	272	14.3	0.95 (0.84-1.07)	0.91 (0.80-1.03)
Current (≤125 days)	7,223	813	11.3	0.74 (0.69-0.79)	0.73 (0.67-0.79)
Current (≤125 days) supplementary adjusted model for 2001-2009	6,883	788	11.4	0.77 (0.71-0.83)	0.76 (0.70-0.83)

^a^Adjusted for age, sex, calendar period, marital status, urbanization of place of residence, any recent surgical procedure, 20 different comorbidities, alcoholism-related conditions, and 19 different preadmission medications. The supplementary model for 2001-2009 is also adjusted for receipt of current year's influenza vaccine, dementia, social medicine-related consultations, application for reimbursement for chronic and terminal illness, conversational therapy, and preventive consultations and services. CI, confidence interval; HR, hazard ratio.

### Additional analyses

**Figure 1:**
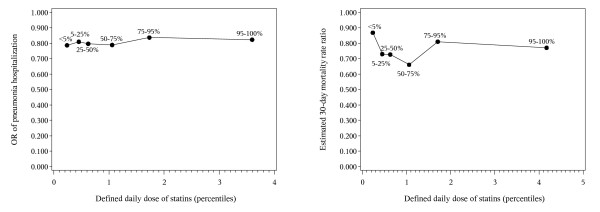
**Statin dose and pneumonia risk and mortality**. Dose-response associations between the number of defined daily doses (DDDs) of statins per day received within 125 days of the admission/index date and pneumonia hospitalization odds ratios **(a) **and hazard ratios for death **(b)**. The relationship between DDDs and pneumonia was assessed by dividing DDDs into percentiles of users. OR, odds ratio.

The results of additional subgroup analyses are presented in Table 5 (case-control and cohort study). In general, the risk and mortality reduction associated with current statin use was observed in most subgroups. However, in some strata, former statin use was also associated with some beneficial effect compared with never use, suggesting either residual confounding by healthy-user effects or a protracted long-term 'legacy' effect of statins. Finally, we found no clear dose-response relationship between statin dose and pneumonia risk (Figure 1a) and 30-day mortality (Figure 1b), respectively. As Figure 1b shows, consumption of approximately 1 statin DDD was associated with the lowest mortality.

**Table 5:** Statin use and pneumonia risk and mortality within various patient subgroups

Variables	Risk of hospitalization for pneumonia, OR (95% CI)		30-day mortality after hospitalization for pneumonia, HR (95% CI)	
Main model for 1997-2009	Current statin use	Former statin use	Current statin use	Former statin use
Calendar time				
1997-2000	0.77 (0.68-0.88)	1.12 (0.84-1.49)	0.57 (0.38-0.85)	1.04 (0.52-2.10)
2001-2004	0.79 (0.74-0.85)	0.88 (0.77-1.00)	0.70 (0.59-0.83)	0.95 (0.70-1.29)
2005-2009	0.81 (0.77-0.84)	0.98 (0.92-1.05)	0.74 (0.67-0.82)	0.90 (0.78-1.03)
Charlson comorbidity index score				
Low, 0	0.78 (0.72-0.84)	1.03 (0.90-1.18)	0.73 (0.56-0.94)	0.77 (0.48-1.22)
Medium, 1-2	0.74 (0.70-0.78)	0.83 (0.76-0.91)	0.73 (0.64-0.83)	0.79 (0.63-0.98)
High, ≥3	0.74 (0.68-0.81)	0.90 (0.79-1.04)	0.69 (0.61-0.77)	0.93 (0.79-1.09)
Age				
15-39 years	0.98 (0.60-1.60)	0.82 (0.42-1.60)	0.00	0.00
40-64 years	0.78 (0.72-0.84)	1.07 (0.95-1.21)	0.77 (0.61-0.97)	0.89 (0.65-1.21)
65-79 years	0.74 (0.71-0.78)	0.91 (0.84-0.99)	0.69 (0.61-0.78)	0.85 (0.71-1.02)
≥80 years	0.80 (0.75-0.85)	0.85 (0.76-0.95)	0.75 (0.66-0.85)	0.95 (0.77-1.16)
Patients with indication for statin use^a^	0.70 (0.67-0.73)	0.78 (0.72-0.85)	0.70 (0.64-0.77)	0.86 (0.75-1.00)
Supplementary adjusted model for 2001-2009				
Receipt of current year's influenza vaccine before index date	0.78 (0.72-0.85)	0.89 (0.78-1.01)	0.76 (0.65-0.88)	0.92 (0.72-1.18)
Preventive GP consultations and services	0.77 (0.69-0.86)	1.10 (0.94-1.29)	0.80 (0.66-0.97)	0.92 (0.70-1.19)
Social medicine-related GP consultations	0.73 (0.66-0.81)	0.91 (0.77-1.08)	0.82 (0.72-0.94)	0.96 (0.79-1.18)
GP conversational therapy	0.80 (0.61-1.04)	0.87 (0.59-1.29)	0.89 (0.67-1.19)	0.66 (0.41-1.05)
Application for reimbursement for chronic illness	0.43 (0.23-0.81)	0.43 (0.16-1.22)	0.64 (0.47-0.86)	0.86 (0.54-1.35)

^a^Previous diagnosis of ischemic or unspecified stroke, ischemic heart disease, atherosclerosis, or diabetes mellitus. For *International Classification of Diseases *(ICD) codes, see Additional file 4. Adjusted pneumonia hospitalization odds ratios (ORs) (case-control study) and 30-day mortality hazard ratios (HRs) (cohort study) associated with current and former use of statins are shown. CI, confidence interval; GP, general practitioner.

## Discussion

In this hitherto largest case-control study on statin use and risk of pneumonia, current statin use was associated with a reduced risk of hospitalization for community-acquired pneumonia. Moreover, we confirmed our previous finding of lower 30-day mortality following hospitalization with pneumonia among current statin users. Not only did pneumonia prognosis improve with statin use, but the risk of hospitalization for pneumonia was lower. This finding suggests that the improved prognosis in statin users was not due simply to surveillance bias resulting in a higher chance of being hospitalized for pneumonia, including milder pneumonia episodes. Our results are consistent with four published case-control studies of pneumonia risk in statin users compared with non-users [10-13]. All four studies used primary-care data and showed ORs ranging from 0.47 to 0.78 in favor of statin use.

In 2009, Dublin and colleagues [14] published a case-control study of 1,125 cases and 2,235 controls in a population of immunocompetent, community-dwelling group health members from 65 to 94 years old. The study showed that statin use was associated with an increased risk of pneumonia. Their baseline-adjusted model (age, sex, and calendar year) yielded an OR of 1.13 (95% CI 0.95 to 1.34), which was very close to our OR (1.17) matched for age, sex, urbanization of place of residence, and index date; the fully adjusted model (comorbid illnesses, lifestyle, demographic characteristics, and functional and cognitive status) yielded an OR of 1.26 (95% CI 1.01 to 1.56). For cases leading to hospital admission (35% of cases), the risk conferred by statin use was even greater (adjusted OR = 1.61, 95% CI 1.08 to 2.39). Confounder data, obtained in part from medical record reviews, included information on functional and cognitive status. This information was used to explain findings and to suggest a strong unaccounted healthy-user effect in previous studies. However, their study was restricted to a selected group of older people. In our study, there was nothing to suggest that patients who used statins had a covariate profile associated with a good prognosis. The age- and sex-matched ORs for pneumonia risk and 30-day mortality associated with statin use were changed from harm to benefit after adjusting for the types of potential confounders included in the analysis carried out by Dublin and colleagues. This discrepancy may be due to differences in the available information on potential confounders or may indicate that the potential healthy-user effect associated with statin use is weaker in our Danish study population.

Although most studies have reported a substantial protective effect of statin use on pneumonia outcomes [16-22], the possibility of a healthy-user effect among statin users in hospitalized patients with pneumonia was proposed on the basis of a 2006 study by Majumdar and colleagues [23] that reported negative statin effects on outcomes. The limitations of this study, including the composite endpoint and the potential overadjustment for covariates (for example, pneumonia severity), have been discussed elsewhere [5,26]. A recent prospective cohort study of 1,895 hospitalized patients with pneumonia in the US also reported some evidence of a healthy-user bias but no decreased risk of severe sepsis (relative risk = 0.98, 95% CI 0.71 to 1.36) or material reduction of 90-day mortality in preadmission statin users (relative risk = 0.90, 95% CI 0.63 to 1.29) [24]. However, the study suggested that continued inpatient statin therapy may reduce pneumonia mortality (adjusted OR = 0.73, 95% CI 0.47 to 1.13) [24], a finding supported by results from Rothberg and colleagues [22] (adjusted OR = 0.90, 95% CI 0.82 to 0.99) and Vandermeer and colleagues [21] (adjusted OR = 0.59, 95% CI 0.38 to 0.92). Unfortunately, we did not have inpatient statin data. Hopefully, future trials will clarify whether continuation or initiation of statin therapy on hospital admission for pneumonia may improve outcome.

To date, there have been no randomized controlled trials large enough to determine the clinical efficacy of statins in patients with pneumonia. In a randomized controlled trial of 83 patients with suspected or documented bacterial infection, statin therapy was associated with a reduction in inflammatory cytokines, but the study was too small to address clinical endpoints [34]. Recently, *post hoc *analyses from the large ASCOT-LLA (Anglo-Scandinavian Cardiac Outcomes Trial-Lipid Lowering Arm) randomized controlled trial of patients with hypertension and additional cardiovascular risk factors suggested a protracted long-term legacy effect after atorvastatin treatment (median of 3.3 years), resulting in reduced long-term all-cause mortality 8 years after closure of the trial (HR = 0.85, 95% CI 0.73 to 0.99). Interestingly, an important contributor to the decreased all-cause mortality was a reduction in deaths from infection and respiratory illness [35].

The main strengths of our study include its population-based design and setting in a tax-supported universal health-care system free at the point of delivery, which reduce the potential for selection and referral bias. The positive predictive value of the DNRP with regard to pneumonia diagnosis is estimated to be 90% (95% CI 82% to 95%) [36], and the degree of completeness of the Danish prescription registries is excellent for reimbursed prescription drugs [37].

A possible study limitation is the use of filled prescriptions as a proxy for actual drug use. However, compliance with statin treatment in Denmark is good [38]. In addition, our observational study design may be vulnerable to uncontrolled confounding. As discussed above, a key question is whether statin users are healthier than non-users in terms of general health factors. Importantly, we found higher rates of most pneumonia risk and prognostic factors among statin users compared with non-users in our analyses, including lifestyle-related diseases such as chronic pulmonary disease, cancer, and diabetes. We adjusted comprehensively for potential confounders, including a wide range of premedication drugs and comorbidities, recent surgery, living with small children, and socioeconomic indicators. To further assess possible residual confounding arising from a healthy-user effect, we retrieved information from the National Health Insurance Service Registry on influenza vaccination and markers for frailty and health awareness. This information, gleaned from GP consultations, is not available in most health registries. After these factors were taken into account, statin use was still associated with a clinically important reduction in pneumonia hospitalizations and deaths. Moreover, the prevalence of current statin use among Danish patients with pneumonia has more than doubled, from an average of 4.6% in 1997-2004 to 10.2% in the updated study period (1997-2009), and statins have become an inexpensive treatment; both factors are likely to dilute any healthy-user effect. Finally, recent data from Denmark showed no indication of a particularly healthy lifestyle associated with statin use. In a questionnaire-based public health survey conducted in the central region of Denmark in 2006, people who used statins were more obese, exercised less, ate healthy foods more, and smoked and drank approximately the same amount as non-users [39].

## Conclusions

Current statin use is associated with a clearly decreased risk of hospitalization for pneumonia and lower 30-day mortality following pneumonia. These results add to the evidence that treatment with statins may have a beneficial effect in reducing severity and possibly preventing pneumonia. Hospitalization rates for pneumonia and sepsis are currently rising in aging Western populations, and case fatality remains high. Our results should be confirmed in randomized statin therapy trials among patients with pneumonia and in statin prevention trials among individuals at risk for pneumonia since positive results would have important clinical and public health implications.

## Key messages

• Statins exert beneficial pleiotropic effects that may decrease risk and improve outcomes of severe bacterial infections like sepsis and pneumonia.

• While awaiting large clinical trials that address this issue, we conducted the hitherto largest combined population-based case-control and cohort study, taking advantage of high-quality Danish health registries and using extensive adjustment for potential confounders.

• Current statin use was associated with a decreased risk of hospitalization for pneumonia and lower 30-day mortality following pneumonia.

## Abbreviations

CI: confidence interval; CRS: Civil Registration System; DDD: defined daily dose; DNRP: Danish National Registry of Patients; GP: general practitioner; HR: hazard ratio; ICD: *International Classification of Diseases*; OR: odds ratio.

## Competing interests

The authors declare that they have no competing interests.

## Authors' contributions

AGN helped to conceive the study idea, to design the study, to analyze the data, and to review the literature and wrote the first draft. HTS helped to conceive the study idea, to design the study, and to collect the data. SPJ helped to conceive the study idea and to design the study. RWT helped to conceive the study idea, to design the study, to analyze the data, and to review the literature. RBN and AHR helped to design the study, to collect the data, and to analyze the data. All authors interpreted the findings and read and approved the final manuscript.

## Supplementary Material

Additional file 1 **Appendix 1**. International Classification of Diseases (ICD)-8 and 10 codes, used to identify pneumonia patients and comorbidities. Click here for file

Additional file 2 **Appendix 2**. Anatomical Therapeutic Chemical (ATC) prescription codes, used to identify statins and other preadmission medications. Click here for file

Additional file 3 **Appendix 3**. Service codes from the Danish National Health Insurance Service Registry, used to identify markers of frailty and health awareness. Click here for file

Additional file 4 **Appendix 4**. Anatomical Therapeutic Chemical (ATC) prescription codes, used to identify patients with indication for statin use (Table 5). Click here for file
