# Supplementary material for: The impact of statin use on pneumonia risk and outcome: a combined population-based case-control and cohort study
Source: Crit Care. 2012 Jul 12;16(4):R122. doi: 10.1186/cc11418 (PMC3580701; doi:10.1186/cc11418)
Supplement: Additional file 1 — Appendix 1. International Classification of Diseases (ICD)-8 and 10 codes, used to identify pneumonia patients and comorbidities. [file cc11418-S1.DOCX]

**International Classification of Diseases (ICD)-8 and 10 codes, used to identify pneumonia patients and comorbidities.**

**1. Pneumonia**

ICD-8: 471, 073, and 480-486

ICD-10: A481, A709, and J12-J18

**2. Charlson Comorbidity Index and comorbidity groups.**

|  | Charlson comorbidity category | ICD-8 | ICD-10 | Score |
| --- | --- | --- | --- | --- |
| 1 | Myocardial infarction | 410 | I21;I22;I23 | 1 |
| 2 | Congestive heart failure | 427.09; 427.10; 427.11; 427.19; 428.99; 782.49 | I50; I11.0; I13.0; I13.2 | 1 |
| 3 | Peripheral vascular disease | 440; 441; 442; 443; 444; 445 | I70; I71; I72; I73; I74; I77 | 1 |
| 4 | Cerebrovascular disease | 430-438 | I60-I69; G45; G46 | 1 |
| 5 | Dementia | 290.09-290.19; 293.09 | F00-F03; F05.1; G30 | 1 |
| 6 | Chronic pulmonary disease | 490-493; 515-518 | J40-J47; J60-J67; J68.4; J70.1;  J70.3; J84.1; J92.0; J96.1; J98.2; J98.3 | 1 |
| 7 | Connective tissue disease | 712; 716; 734; 446; 135.99 | M05; M06; M08; M09;M30;M31;  M32; M33; M34; M35; M36; D86 | 1 |
| 8 | Ulcer disease | 530.91; 530.98; 531-534 | K22.1; K25-K28 | 1 |
| 9 | Mild liver disease | 571; 573.01; 573.04 | B18; K70.0-K70.3; K70.9; K71; K73; K74; K76.0 | 1 |
| 10 | Diabetes type 1    Diabetes type 2 | 249.00; 249.06; 249.07; 249.09  250.00; 250.06; 250.07; 250.09 | E10.0, E10.1; E10.9  E11.0; E11.1; E11.9 | 1 |
| 11 | Hemiplegia | 344 | G81; G82 | 2 |
| 12 | Moderate to severe renal disease | 403; 404; 580-583; 584; 590.09; 593.19; 753.10-753.19; 792 | I12; I13; N00-N05; N07; N11; N14; N17-N19; Q61 | 2 |
| 13 | Diabetes with end organ damage  Type 1  Type 2 | 249.01-249.05; 249.08  250.01-250.05; 250.08 | E10.2-E10.8  E11.2-E11.8 | 2 |
| 14 | Any tumor | 140-194 | C00-C75 | 2 |
| 15 | Leukemia | 204-207 | C91-C95 | 2 |
| 16 | Lymphoma | 200-203; 275.59 | C81-C85; C88; C90; C96 | 2 |
| 17 | Moderate to severe liver disease | 070.00; 070.02; 070.04; 070.06; 070.08; 573.00; 456.00-456.09 | B15.0; B16.0; B16.2; B19.0; K70.4; K72; K76.6; I85 | 3 |
| 18 | Metastatic solid tumor | 195-198; 199 | C76-C80 | 6 |
| 19 | AIDS | 079.83 | B21-B24 | 6 |

**3. Additional comorbidities**:

Alcoholism-related disorders

ICD-8: 291, 303, 979, 980, 57710

ICD-10: DF10, DG312, DG621, DG721, DI426, DK292, DK860, DR780, DT51, DZ721

And/or

ATC N07BB (Disulfiram)

Obesity

ICD-8: 27799

ICD-10: DE65, DE66

Any surgical procedure within 90 days of index date

All procedure codes = K as the first letter. http://medinfo.dk/sks/brows.php?s_nod=32268
