# Supplementary material for: The impact of statin use on pneumonia risk and outcome: a combined population-based case-control and cohort study
Source: Crit Care. 2012 Jul 12;16(4):R122. doi: 10.1186/cc11418 (PMC3580701; doi:10.1186/cc11418)
Supplement: Additional file 2 — Appendix 2. Anatomical Therapeutic Chemical (ATC) prescription codes, used to identify statins and other preadmission medications. [file cc11418-S2.DOCX]

**Anatomical Therapeutic Chemical (ATC) prescription codes, used to identify statin and other preadmission medications.**

Simvastatin: C10AA01, B04AB01

Atorvastatin: C10AA05

Pravastatin: C10AA03

Other statins: C10AA0X, not included in other categories.

Oral steroids: H02AB

Other immunosuppressive drugs: L01, L04

Inhaled beta2-agonists only: R03AC, R03AK03, R03AK04

Inhaled corticosteroid therapy only: R03BA

Inhaled corticosteroid therapy and beta2-agonists combination: R03AK06, R03AK07

Beta blockers: C07

Low-dose aspirin: B01AC06

Loop diuretics: C03C

Thiazides: C03A

Angiotensin-converting enzyme inhibitor: C09A, C09B, C08DA51

ATII antagonists: C09C, C09D

Digoxin: C01AA

Vitamin K antagonist: B01AA

Calcium antagonists: C08

Nitrates: C01DA

Antipsychotics: N05A, except N05AN

Nonsteroidal anti-inflammatory drugs: M01A

Proton pump inhibitors: A02BC

Systemic antibiotic therapy: J01
