# Supplementary material for: The impact of statin use on pneumonia risk and outcome: a combined population-based case-control and cohort study
Source: Crit Care. 2012 Jul 12;16(4):R122. doi: 10.1186/cc11418 (PMC3580701; doi:10.1186/cc11418)
Supplement: Additional file 3 — Appendix 3. Service codes from the Danish National Health Insurance Service Registry, used to identify markers of frailty and health awareness. [file cc11418-S3.DOCX]

**Service codes from the Danish National Health Insurance Service Registry*** **(sygesikringsregistret), used to identify markers of frailty and health awareness.**

Receipt of current year’s reimbursed influenza vaccine before index date: 8920-8925, 8935-8938, 9820, 9821, 8926-8932, 8934

Preventive GP consultations and services: 0103, 0106, 4004, 4088, 4031, 4032-4034

Social medicine-related GP consultations: 3000, 3201, 3301-3304, 4530

GP conversational therapy: 6101, 6000, 6100, 4003, 4050, 4063, 4106, 4021-4027

Application for reimbursement for chronic illness: 5090, 5091-5092, 5991-5992

Application for reimbursement for terminal illness: 5093, 5993

GP diagnosis and treatment of dementia: 2602, 2603, 4049, 4131-4135, 4467, 4546

*<http://www.sst.dk/Indberetning%20og%20statistik/Sundhedsstyrelsens%20registre/Sygesikringsregister.aspx>

Click “ydelsesoversigt” (service codes = ydelseskoder).
