# Supplementary material for: The impact of statin use on pneumonia risk and outcome: a combined population-based case-control and cohort study
Source: Crit Care. 2012 Jul 12;16(4):R122. doi: 10.1186/cc11418 (PMC3580701; doi:10.1186/cc11418)
Supplement: Additional file 4 — Appendix 4. Anatomical Therapeutic Chemical (ATC) prescription codes, used to identify patients with indication for statin use (Table 5). [file cc11418-S4.DOCX]

**Anatomical Therapeutic Chemical (ATC) prescription codes, used to identify patients with indication for statin use (Table 5).**

Diabetes: A10A, A10B, 249, 250, DE10, DE11, DE12, DE13, DE14, DH360, DO24, not DO244

Stroke: 433, 434, DI63, DI64, DI693, DI694, DI698

Ischemic heart disease: 410–414, DI20-DI25

Peripheral atherosclerosis: 440, DI70
